# Defining the Subcellular Interface of Nanoparticles by Live-Cell Imaging

**DOI:** 10.1371/journal.pone.0062018

**Published:** 2013-04-26

**Authors:** Peter H. Hemmerich, Anna H. von Mikecz

**Affiliations:** 1 Leibniz-Institute for Age Research, Fritz-Lipman-Institute, Jena, Germany; 2 IUF - Leibniz Research Institute for Environmental Medicine at Heinrich-Heine-University Duesseldorf, Duesseldorf, Germany; Brandeis University, United States of America

## Abstract

Understanding of nanoparticle-bio-interactions within living cells requires knowledge about the dynamic behavior of nanomaterials during their cellular uptake, intracellular traffic and mutual reactions with cell organelles. Here, we introduce a protocol of combined kinetic imaging techniques that enables investigation of exemplary fluorochrome-labelled nanoparticles concerning their intracellular fate. By time-lapse confocal microscopy we observe fast, dynamin-dependent uptake of polystyrene and silica nanoparticles via the cell membrane within seconds. Fluorescence recovery after photobleaching (FRAP) experiments reveal fast and complete exchange of the investigated nanoparticles at mitochondria, cytoplasmic vesicles or the nuclear envelope. Nuclear translocation is observed within minutes by free diffusion and active transport. Fluorescence correlation spectroscopy (FCS) and raster image correlation spectroscopy (RICS) indicate diffusion coefficients of polystyrene and silica nanoparticles in the nucleus and the cytoplasm that are consistent with particle motion in living cells based on diffusion. Determination of the apparent hydrodynamic radii by FCS and RICS shows that nanoparticles exert their cytoplasmic and nuclear effects mainly as mobile, monodisperse entities. Thus, a complete toolkit of fluorescence fluctuation microscopy is presented for the investigation of nanomaterial biophysics in subcellular microenvironments that contributes to develop a framework of intracellular nanoparticle delivery routes.

## Introduction

The fact that natural and engineered nanoparticles (NPs) have novel properties and the potential to access isolated parts of the body, including the brain, creates a coherent interest to develop nanomaterials for biomedical applications such as imaging, diagnostics and therapeutics. At their target organ, NPs effectively enter cells by endocytosis [1]–[4], although the underlying in-depth mechanisms of cross-membrane translocation are still largely unknown. The biological behavior of NPs depends primarily on how they interface to biomolecules and their surroundings [5]. Since the surface of NPs critically determines their interaction with biomolecules, elaborate methods are developed to engineer specific NP-bio-interactions by modulation of particle surfaces [6], [7]. Consistent with this, understanding the biological interface of nanomaterials requires biophysical methods to analyse the properties and behavior of NPs within cells [8].

NPs are generally defined by their size, chemical composition, morphology, surface area, surface chemistry, and reactivity in solution. State of the art methods to analyse these NP-characteristics include transmission electron (TEM) and atomic force microscopy that provide information about particle size, morphology, and to a certain extend surface chemistry. More chemical information is yielded by Tip-Enhanced Raman Spectroscopy that increases sensitivity down to the single molecule level. Characterization of NPs in solution conventionally involves analyses of features such as hydrodynamic radius (*R_h_*) and zeta potential by means of dynamic light scattering.

However, as all these methods represent *in vitro* tools, e.g. they are not suitable for analysis of physicochemical NP-properties in the context of the living cell. After translocation over the cell membrane via pinocytosis [9], [10] NPs reach the cytoplasm that constitutes a crowded molecular environment [11]. It contains a plethora of macromolecules organized as proteins, nanomachines, protein complexes, vesicles, and organelles [12]. Thus, transient or stable non-covalent interactions occur between the surface of NPs and intracellular macromolecules. A protein 'corona' builds in the cytoplasm, becomes a major element of the biological identity of the respective NP [13], and is likely to change NP-properties such as hydrodynamic radius (*R_h_*) and surface chemistry. While a detailed characterization of the intracellular protein corona of NPs remains a challenging task due to lack of adequate techniques, highly variable NP-bio-interactions were identified that seem to correlate with specific particle properties. Once polyethyleneimine NPs are internalized into the cytoplasm they escape endosomal vesicles and are transported in a microtubule-dependent manner to the perinuclear region within 10 minutes [14]. While quantum dots and many other NPs remain associated with lysosomes and endosomal compartments for days [15], interactions of cationic polystyrene (PS) NPs in the cytoplasm are characterized by lysosomal rupture, mitochondrial damage and induction of toxic oxidative stress that starts after less than 1 hour of nanomaterial addition [16], [17]. But the NP-bio-interface is not restricted to the cytoplasm. Silica NPs penetrate into the cell nucleus, where they induce formation of amyloid-like protein inclusions, and inhibition of gene expression that correlates with a significant reduction of cell proliferation, and manifestation of cellular senescence [18], [19].

While different types of particles and cellular systems were applied in these prototypic reports, the results suggest that NPs may exert many of their intracellular effects directly, e.g. dependent on their subcellular localization and interaction. Thus, further understanding of the underlying mechanisms requires analyses of physicochemical NP-properties in the living cell [8]. In order to define intracellular NP-bio-interactions we adopted a combination of protocols for biophysical characterization of fluorescently labelled NPs, in particular, fluorescence recovery after photobleaching (FRAP) [20], fluorescence correlation spectroscopy (FCS) [21], [22], and raster image correlation spectroscopy (RICS) [23]. This combination of quantitative fluorescence fluctuation microscopy techniques enabled analysis of the behavior of fluorescently labelled carboxylated (COOH)-PS or plain-PS NPs as well as silica NPs in the cytoplasm and cell nucleus at an unprecedented spatio-temporal resolution. At distinct NP-biomolecule interfaces we describe quantitatively in live cells (i) fast, dynamin-dependent uptake of NPs via the cell membrane within seconds, resembling the time kinetics of endocytosis at nerve terminals [24], (ii) rapid on/off-binding dynamics at cytoplasmic organelles such as mitochondria, and (iii) rapid nuclear translocation. By FCS and RICS, e.g. determination of hydrodynamic radii of the particles, we show that these intracellular interactions are highly dynamic and performed by monodisperse NPs.

## Results and Discussion

### Size of nanoparticles

**Figure 1:**
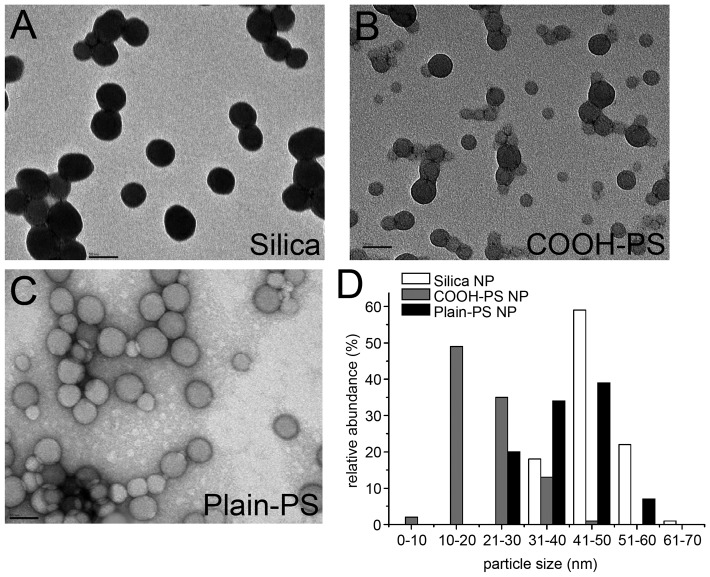
TEM analysis of fluorescent nanoparticles. Fluorescent (A) silica, (B) carboxylated polystyrene (COOH-PS [YO]) and (C) plain polystyrene (plain-PS [YG]) nanoparticles (NPs) were visualized by transmission electron microscopy (TEM) at 250K-fold magnification. From a series of randomly chosen TEM images, particle size was quantitated (n>200 each) and the relative abundance plotted against different classes of particle size/diameter (D). The mean diameter of silica, COOH-PS [YO] and plain-PS [YG] NPs was 46±7 nm, 22±7 nm and 45±5 nm, respectively. Bars, 50 nm.

It was demonstrated previously that a variety of polystyrene (PS) and silica NPs enter cells in culture [17]–[19], the gut lumen or tissue of the model organism *Caenorhabditis elegans* [25] (Scharf and von Mikecz, unpublished results), and the lung tissue of factory workers [26]. In order to define the properties of particles used in the current analyses, transmission electron microscopy (TEM) was employed to determine the size of PS NPs and silica NPs in detail. By means of TEM silica NPs (coupled with the fluorochrome FITC, Figure 1A) and COOH-PS [coupled with the fluorochrome yellow orange, YO] NPs (Figure 1B) appeared monodisperse or in small clusters. The small NP-clusters contained between two and eight particles, but never more than ten. Large particle agglomerates were not observed. Plain-PS [coupled with the fluorochrome yellow green, YG] NPs were likewise monodisperse or in small clusters (Figure 1C). These data confirm previous observations that PS nanospheres predominately occur as monodisperse entities under aqueous conditions [16]. Silica NPs range in size from 30 nm to 60 nm with a mean diameter of 46±7 nm. COOH-PS [YO] NPs constitute a mixture of heterogeneously sized populations ranging from 10 nm to 50 nm with a mean size of 22±7 nm. Plain-PS [YG] NPs had an average size of 45±5 nm (Figure 1D, n>200). Consistent with the paradigm that NPs with a diameter between 30 and 60 nm are preferentially taken up by cultured cells in contrast to their smaller or larger counterparts [4], [27], the diameter analyses by TEM and dynamic light scattering (Table S1) warrant that NPs used in this study are suited to investigate intracellular particle-bio-interactions.

### Subcellular distribution of nanoparticles in living cells

**Figure 2:**
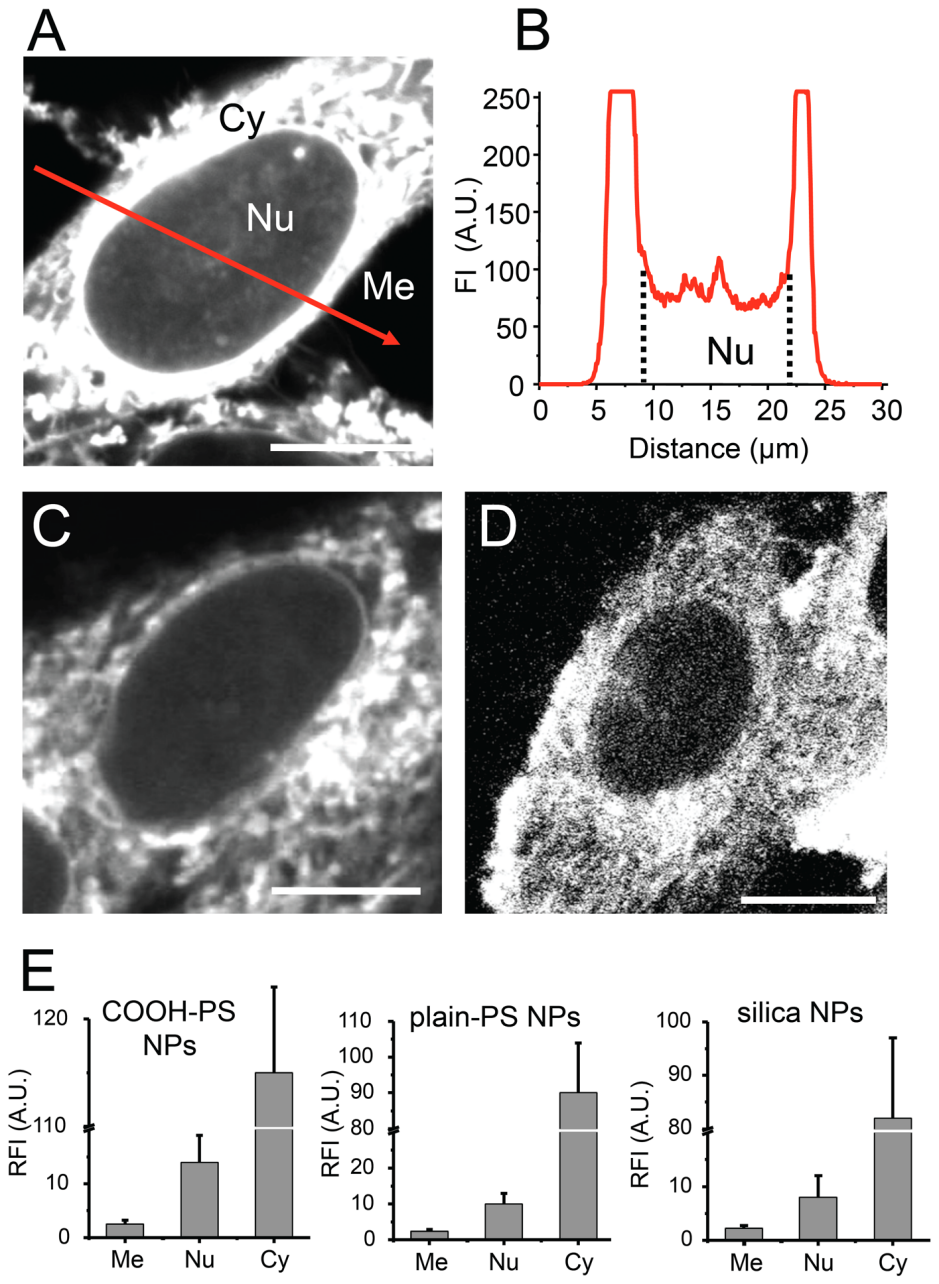
Cellular distribution of nanoparticles. (A) Living HEp-2 cells were incubated with fluorescent (A) COOH-PS [YO] , (C) plain-PS YG or (D) silica NPs for one hour and analyzed by confocal microscopy. Representative micrographs show mid-nuclear confocal sections detecting NP fluorescence in the nucleus (Nu), the cytoplasm (Cy), and in the medium (Me). (B) Fluorescence intensity of COOH-PS [YO] NPs was recorded along the red line and displayed. Bars, 10 Çm. (E) Quantification of fluorescent NP-accumulation in the cytoplasm and the nucleus of living cells. HEp-2 cells were incubated with the indicated NPs for 1 hour. Mean fluorescence intensities were determined in circular regions (2 Çm in diameter) in the medium, nucleus or cytoplasm. Graphs show relative fluorescence intensities (RFI) mean values and standard deviations (n = cells each) normalized to the fluorescence intensity in the medium which was set to 2. A.U., arbitrary units.

To quantitatively analyze the subcellular localization of NPs, living HEp-2 cells were incubated with fluorescent COOH-PS [YO], plain-PS [YG] and silica NPs for one hour, followed by confocal imaging of the fluorescence signals. COOH-PS [YO] NPs strongly accumulate in the cytoplasm as described before, however additional fluorescence is observable in the cell nucleus as a diffuse pattern (Figure 2A, Nu). Corresponding line scan fluorescence measurements through mid-nucleus confocal sections confirm that a substantial amount of NP-fluorescence is present in the nucleus (Figure 2B). Similar cellular distribution patterns are obtained for fluorescent plain-PS [YG] NPs (Figure 2C) and silica NPs (Figure 2D). Quantification of relative fluorescence intensities shows that all NPs analyzed here, accumulate in the cytoplasm as well as in the nucleus of living cells after one hour (Figure 1E). Note, that generally the mean particle fluorescence intensity in the cytoplasm is at least one order of magnitude stronger than in the nucleus. Thus, detection of fluorescent NPs in the nucleus usually requires imaging settings which inevitably produce over-saturated NP fluorescence patterns in the cytoplasm (Figures 2A and B). The results indicate that silica and PS NPs with different surface coatings enter living cells rapidly and throughout cellular compartments, e.g. that a particle-subfraction translocates into the nucleus. The latter observation confirms the cell nucleus as a target for NPs, and expands the list of nanomaterials with nuclear localization [16], [18], [28]–[30].

In the cytoplasm, silica and PS NPs accumulate at reticular as well as vesicular structures, and in the perinuclear region (Figure 2 and Figure S1 A). Colocalization analyses show that the cytoplasmic distribution pattern of COOH-PS [YO] NPs is distinct from that of plain-PS [YG] NPs (Figure S1 A,B). The reticulated localization pattern of COOH-PS [YO] NPs resembles the intracellular distribution of mitochondria. In confirmation, a colocalization analysis with Mitotracker showed preferential concentration of COOH-PS [YO] NPs at mitochondrial microenvironments (Figure S1 C). In contrast, plain-PS NPs distribute in a filamentous localization pattern and additionally accumulate at vesicle-like structures during interphase and mitosis (Figure S1 A,B). Neither plain-PS [YG] nor COOH-PS [YO] NPs colocalized with the lysosomal marker DQ ovalbumin or the Golgi marker Brefeldin A (data not shown). It is widely acknowledged that the surface composition of NPs contributes to their localization in the cytoplasm. Thus, the observed differences might result from nano-bio-interactions of diverse PS NPs with distinct cytoplasmic structures. In the case of COOH-PS [YO] as well as plain-PS [YG] NPs these interactions with cytoplasmic structures persist throughout and do not interfere with mitosis (Figure S1 D, and data not shown). Confirmedly, cell viability analyses showed no reduction, and markers for apoptotic cell death such as cleavage products of caspases remained negative for at least 48 hours after exposition with COOH-PS NPs (von Mikecz, unpublished results).

### Nanoparticle dynamics in living cells as measured by FRAP

**Figure 3:**
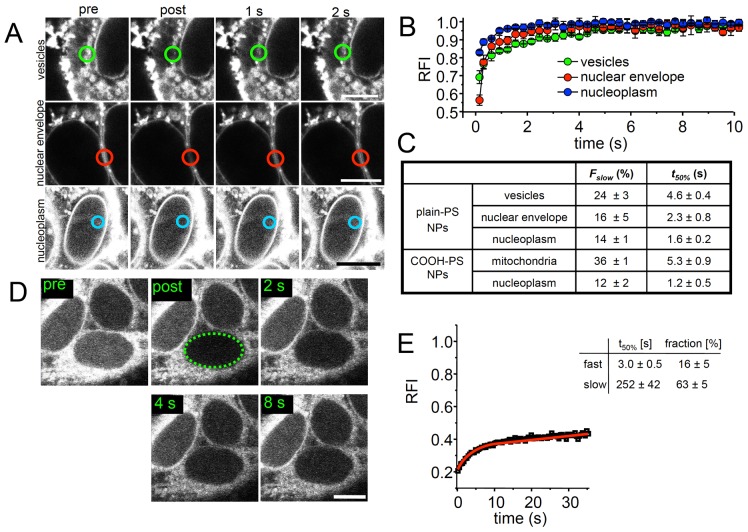
Quantitative analysis of PS NP dynamics in living cells by FRAP. (A) HEp-2 cells were subjected to fluorescence recovery after photobleaching (FRAP) after incubation with fluorescent plain-PS [YG] NPs for 1 hour. Quantification was performed in circular regions containing cytoplasmic vesicles (green), at the nuclear envelope (red; micrograph of two adjacent cells), or in the nucleoplasm (blue). Note that FRAP analyses in the nucleus required increasing the detector gain of the confocal microscope due to the lower fluorescence signal in this compartment compared to the cytoplasm. Therefore the fluorescence signals in FRAP images appear over-saturated. Bars, 10 µm. (B) FRAP curves from independent experiments (n = 10) were then quantitated by plotting the relative fluorescence intensity (RFI) over time (graphs). Data were normalized to RFI = 1 within the bleached region before the bleach pulse. (C) The amount of slowly moving (interacting) NPs (*F*_slow_) and their recovery halftime (*t*_50%_) at the indicated cellular structures were determined as described in Materials and Methods. (D) Whole nucleus FRAP. COOH-PS [YO] NP-fluorescence was bleached in a region containing the whole nucleus (dotted green line) and fluorescence recovery into the nucleus monitored over time. (E) Quantitation of whole nucleus FRAP experiments. Graph shows mean values from at least ten measurements (standard deviations were below 16% of mean values, not shown). (E) FRAP data were fitted to a two-component exponential function (red graph) from which the recovery halftimes and the relative fractions of fast and slowly exchanging COOH-PS [YO] NP populations were determined. Bars, 10 µm.

We next characterized the nano-bio-interface in living cells by fluorescence recovery after photobleaching (FRAP). HEp-2 cells were incubated with plain-PS [YG] NPs to assess intracellular dynamics of NPs at cytoplasmic vesicles (Figure 3A, green circles), in the perinuclear region, e.g. nuclear envelope (red circles), and in the cell nucleus (blue circles). FRAP revealed rapid fluorescence recovery of plain-PS [YG] NPs at all intracellular structures examined. Quantitation of FRAP curves confirmed rapid fluorescence recovery and showed that fluorescence intensity had returned to pre-bleach values after 10 seconds at cytoplasmic vesicles, 5 seconds at the nuclear membrane and 2 seconds in the nucleoplasm (Figure 3B). The results indicate that plain-PS [YG] NPs rapidly exchange at topologically more immobile subcellular microenvironments such as vesicle membranes, the nuclear envelope or chromatin, respectively.

The FRAP data were fitted to two-component exponential functions as described in Materials and Methods. From the exponential term that describes the slow fraction (*F_slow_*) of NPs, the recovery halftime (*t_50%_*) of plain-PS [YG] NPs for this fraction was derived. The analysis revealed that plain-PS [YG] NPs interact with cellular structures very dynamically with recovery halftimes in the seconds range (Figure 3C). Rapid FRAP in the nucleus without the presence of an immobile fraction also indicates free diffusion properties of PS-NPs throughout the nucleoplasm. The relatively low recovery-halftime (one to 2 seconds) of the slow fraction of NPs in the nucleus suggest that NPs move through the nucleus by free diffusion and random collision with chromatin. FRAP analyses likewise revealed rapid and complete exchange of COOH-PS [YO] NPs at mitochondria within seconds (Figure 3C). Such rapid exchange kinetics suggest fast and transient interactions with molecules of the outer mitochondrial membrane, rather than stable binding to the membrane. Together with TEM results showing that 50 nm COOH-PS NPs do not enter mitochondria [17], the transient nature of interactions at the interface between NPs and the mitochondrial membrane might explain previous observations that COOH-PS NPs, in contrast to cationic PS nanospheres, neither induce mitochondrial injury [16], [17], nor reduce cell viability. Assuming that particle dynamics as measured by kinetic imaging are of specific and consistent nature, systematic FRAP-analysis of interactions between NPs and intracellular structures has the potential to evolve into a framework for prediction of both nano-bio-interaction and toxicity. While the present study exemplary shows FRAP with PS NPs, such analyses are applicable on virtually all fluorescently labelled nanomaterials and biomedical problems.

To address the dynamics of nuclear import we performed FRAP of COOH-PS [YO] NPs within the entire nucleus (Figure 3D,E). This approach is suitable to yield information on the nucleocytoplasmic exchange rates of molecules [31]. Quantitation of whole nucleus bleach experiments revealed a biphasic nuclear NP uptake. Rapid influx of COOH-PS [YO] NPs into the nucleus occurs during the first 5 to 10 seconds after the bleach, followed by a slow, but steady recovery over minutes (Figures 3D, and data not shown). Fitting of FRAP data to two-component exponential functions shows fractions and recovery halftimes of two differently mobile COOH-PS [YO] NP populations that enter the nucleus with distinct dynamics (Figure 3E). The fast population (16±5%, t_50%_ = 3.0±0.5 s) may represent exchange of the smaller particles (10–20 nm) in-and-out of the nucleus by free diffusion through nuclear pores. In contrast, the slow fraction (63±5%, t_50%_ = 251±42 s) is indicative of an active import mechanism, probably of those NPs which are too large for free diffusion through the nuclear pore. The remaining ∼20% of unbleached fluorescence likely results from incomplete bleaching of the nuclear NP pool and/or rapid free diffusion of COOH-PS [YO] NPs smaller than 10 nm that were identified by TEM (Figure 1D). Additionally, the fast COOH-PS [YO] NP population is in agreement with a previous TEM-based study showing that gold NPs with a diameter of up to 39 nm passively translocate from the cytoplasm to the nucleus via nuclear pores [32]. Since FRAP was performed at least 1 hour after NP addition, the data actually describe the traffic of NPs in and out of the nucleus. It is therefore possible that cellular proteins which adhere to the surface of NPs may be subject to aberrant cellular targeting. Consistent with the idea of controlled transfer of nanomaterials to the cell nucleus whole nucleus FRAP analyses as performed in this study may aid to (i) develop NPs able to carry macromolecules via nuclear pores and execute their delivery to nuclear processes, as well as (ii) elucidate nucleocytoplasmic transport of NPs in depth.

### Particle dynamics in living cells as measured by FCS

**Figure 4:**
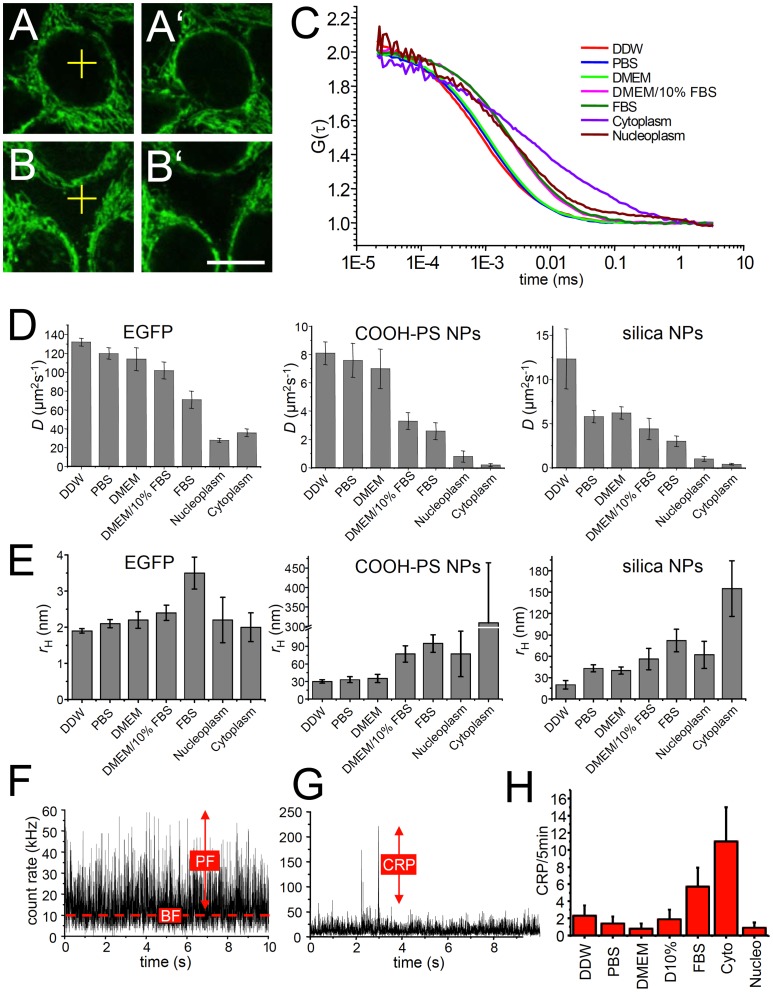
Diffusion behavior of nanoparticles in living cells and *in vitro*. (A–B') Sample micrographs: HEp-2 cells were incubated with fluorescent COOH-PS [YO] NPs for 30 min and FCS measurements were performed in the nucleus (A, yellow cross) or at cytoplasmic locations outside the strongly labelled regions (B, yellow cross). Bar, 10 µm. A‘ and B‘ show the same subcellular locations after the FCS measurement. (C) Autocorrelation curves from FCS measurements at room temperature in distilled water (DDW), phosphate-buffered saline (PBS), Dulbecco‘s modified Eagle‘s medium (DMEM), in DMEM containing 10% fetal calf serum (DMEM/10% FBS), in undiluted FBS, as well as in the nucleoplasm and cytoplasm of living HEp-2 cells. (D) The diffusion coefficients of EGFP, COOH-PS [YO] NPs and silica NPs in different solvents or compartments of living cells were determined by FCS measurements. (E) The hydrodynamic radii of EGFP, COOH-PS [YO] NPs and silica NPs were determined in different solvents or compartments of living cells from the respective diffusion coefficients obtained by FCS. (F) Representative count rate trace of an FCS measurement of COOH-PS [YO] NPs in the nucleus. BF, background fluorescence; kHz, kilo Hertz; PF, particle fluorescence. (G) Representative count rate trace of an FCS measurement of COOH-PS [YO] NPs in the cytoplasm. CRP, count rate peaks. (H) The number of CRPs during 5 minutes FCS measurements was quantified from count rate traces within the indicated solvents or cellular compartments. Cyto, cytoplasm; *D*, diffusion coefficient; NPs, nanoparticles; nucleo, nucleoplasm.

In order to analyse in living cells NP-properties in correlation with NP-dynamics we applied fluorescence correlation spectroscopy (FCS). FCS measurements were performed in the nucleus and the cytoplasm of NP-loaded HEp-2 cells at 25°C (Figure 4A and B, respectively). For comparison, diffusion of NPs was also measured *in vitro* at 25°C in different solutions such as water, phosphate-buffered saline (PBS), Dulbecco's modified essential medium (DMEM), DMEM containing 10% fetal bovine serum (DMEM/10% FBS), and in 100% FBS. Autocorrelation curves obtained from FCS measurements are shown in Figure 4C. The curves were fitted to a one-component-free-diffusion model which yields excellent fits for measurements in water, PBS, DMEM, DMEM/10% FBS, and 100% FBS, and sufficiently good fits for FCS measurements in the cytoplasm or the nucleus of living cells (data not shown). In addition, the diffusion coefficient for free GFP in solution and in living cells was determined (Figure 4D). The diffusion coefficients of fluorescently labelled COOH-PS [YO] and silica NPs in water are 8.1 (±1.1) µm^2^s^−1^ and 12.4 (±2.3) µm^2^s^−1^, respectively, which translate into hydrodynamic radii of 29±4 nm and 17±4 nm, respectively (Figure 4E). These values are in perfect agreement with the TEM data and suggest that a majority of the NPs is monodisperse in water. Notably, the *in vitro* diffusion properties of NPs remained unaffected in PBS and DMEM, however, as soon as proteins were present, as in the case of DMEM/10% FBS, 100% FBS, nucleoplasm or cytoplasm, the diffusion coefficient decreased dramatically (Figure 4D). Conversely, the average hydrodynamic radius of COOH-PS [YO] and silica NPs increased to ∼90 nm in FBS (Figure 4E). The results indicate an increase of NP-size by surface binding of serum proteins or formation of small clusters, or both. Surface binding of serum proteins is in agreement with the idea that a protein 'corona' builds around the particle core in the cytoplasm, and constitutes a major element of the biological identity of respective NPs [13]. While 'corona' formation slows down particle dynamics it may at the same time inhibit intracellular agglomeration of PS-NPs. In contrast to COOH-PS [YO] NPs, we already observed a substantial increase of the hydrodynamic radius of silica NPs when PBS was used as a solvent instead of water (Figure 4E). This observation might be related to a higher capacity of self-interaction of silica NPs compared to COOH-PS NPs in the presence of PBS. A substantial decrease of the *in vitro* diffusion coefficient and increase of hydrodynamic radius in the presence of serum proteins was also observed for soluble green fluorescent protein (GFP), indicating that obstruction of dynamics by interactions with proteins applies for both proteins and NPs (Figure 4D).

The diffusion coefficient of COOH-PS [YO] and silica-NPs in the cell nucleus as determined by FCS was 0.8 (±0.3) µm^2^s^−1^ and 1.2 (±0.3) µm^2^s^−1^, respectively, which equals an approximately ten-fold reduction compared with values obtained in water (Figure 4D). For evaluation of apparent hydrodynamic radii of NPs in living cells, the increased viscosity due to immobile cellular diffusion barriers (organelles, vesicles, filaments, chromatin etc.) must be considered [33]. For inert particles, such as dextran or ficoll in the size range of the NPs used here, viscosity in nuclei of living cells is increased by a factor of four compared to the viscosity in water [34]. Considering the increased viscosity within the cellular microenvironment, the diffusion coefficients measured by FCS yield apparent hydrodynamic radii of COOH-PS and silica NPs in nuclei of ∼60 nm (Figure 4E). From these data we conclude that the NPs studied here diffuse as surface-coated monodisperse particles or small clusters throughout the nuclear volume. In combination FRAP and FCS results imply that NPs might interact with nuclear structures such as chromatin and ribonucleoprotein complexes throughout the nucleoplasm and likely interface with active nuclear processes. Consistent with this idea we showed previously that silica NPs enter the nucleus, induce aberrant protein aggregation, and inhibit replication as well as transcription. As a consequence of nuclear silica NP wash-in normally proliferating cells undergo a permanent cell cycle arrest that resembles cellular senescence [18].

The apparent hydrodynamic radii of COOH-PS and silica NPs in the cytoplasm were ∼300 nm and 150 nm, respectively (Figure 4E). While this result might suggest formation of large NP clusters, we did not observe such large clusters by confocal microscopy that otherwise enables imaging of single, bulk COOH-PS [YG] particles with a diameter of 200 nm (see also subsequent results). Thus, the low diffusion coefficients of NPs in the cytoplasm rather reflect additional diffusion inhibition of monodisperse NPs and small NP clusters imposed by immobile obstacles, such as vesicles and filaments as shown previously [35]–[38]. In addition, unspecific transient binding events to immobile structures in the cytoplasm, as revealed by FRAP (Figure 3) likely contribute to decreased mobility. Our results are in excellent agreement with FCS studies employing similar-sized dextran in the cytoplasm [37], and indicate a higher degree of diffusion obstruction for silica and COOH-PS NPs in the cytoplasm compared with the nucleus. This conclusion is also consistent with observations showing that the effective viscosity for diffusion in the cytoplasm depends on particle size [39]. Indeed, the effective viscosity for larger particles in the cytoplasm can be 6 to 20-fold higher compared to water [35], [36].

### Very low agglomeration propensity of COOH-PS and silica NPs *in vitro* and *in vivo*

The majority of our FCS recordings of fluorescent NPs yielded count rate traces consisting of background fluorescence and signal fluctuations by photons emitted from the freely (or obstructed) diffusing particles (Figure 4F). Occasionally, count rate peaks (CRPs) were observed (Figure 4G, arrow). Such singular events during FCS measurements are indicative of large mobile structures containing many fluorescent molecules [40], [41]. Thus, CRPs during FCS of NPs may represent large clusters or agglomerates. The occurrence of CRPs was quantified from at least three long-time FCS measurements in different solvents or cellular compartments. Low propensity of COOH-PS NP agglomeration (<2 CRPs/5 minutes) occurs in distilled water, PBS, DMEM, DMEM/10%FBS, and the cell nucleus (Figure 4H) corroborating that the NP-interface in these settings mainly consists of monodisperse nanomaterials. An average of 6 or 12 CRPs within five minutes was counted in 100% FBS, or the cytoplasm, respectively. It is important to note that in the cytoplasm COOH-PS NP-loaded organelles (such as mitochondria, Figure S1) moving through the FCS volume during the measurement contribute to CRPs, and cannot be distinguished from particle agglomerates. Similar results concerning the distribution of CRPs in different intracellular compartments were obtained with silica and plain-PS NPs (data not shown). Thus, we conclude that the NPs investigated here rarely agglomerate into large complexes in the environment of a living cell.

### Quantitation of NP dynamics in living cells by RICS

**Figure 5:**
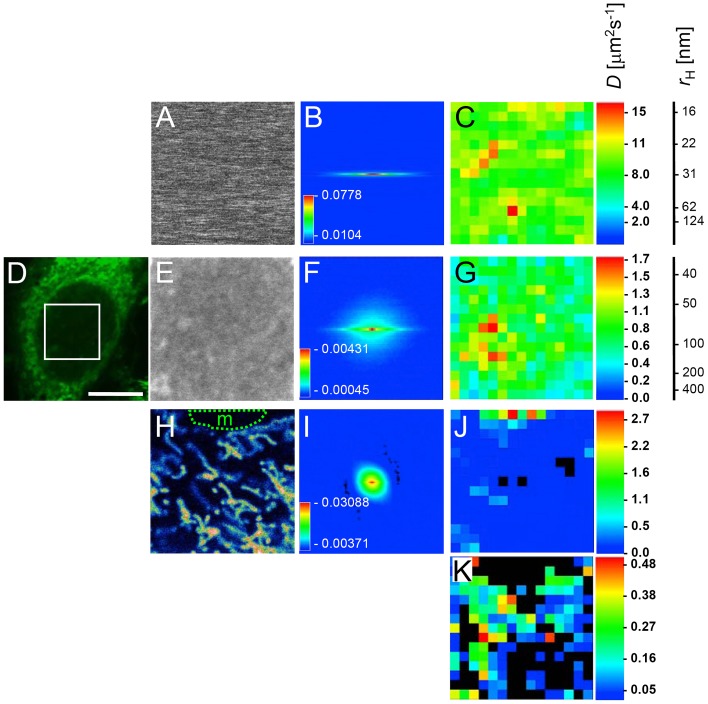
Nanoparticle dynamics in living cells and *in vitro* assessed by RICS. (A) A time series of fluorescent COOH-PS [YO] NPs that at room temperature freely diffuse in water was acquired by confocal microscopy (frame size: 512×512 pixels; pixel size: 0.03 mm; scan speed: 6.4 ms/pixel). (A) shows one image of the time series image stack. (B) Two-dimensional spatial autocorrelation of the image stack displaying the region for 32 pixel shifts in negative and positive directions. Numbers along the color code bar indicate the correlation values. (C) Using a region of interest (ROI, 64x64 pixels) analysis, which scans sub-regions within the original image, a map for the diffusion coefficient is generated along with a diffusion coefficient *(D)* color map. A corresponding scale indicating the hydrodynamic radius (*r*_H_) is also shown. (D) Image of a living HEp-2 cell incubated with fluorescent COOH-PS [YO] NPs for 1 hour. The white box indicates the region were subsequently a confocal time series image stack was acquired (frame size: 512×512 pixels; pixel size: 0.03 mm; scan speed: 6.4 ms/pixel). One image of the resulting image stack is shown in (E). (F) Two-dimensional spatial autocorrelation of the image stack (as described in B). (G) Diffusion coefficient distribution map of the region shown in (E) along with a diffusion coefficient *(D)* color map and a scale indicating the corresponding apparent hydrodynamic radius (*r*_H_) corrected for the apparent viscosity of 30 nm particles in the nucleus [52]. (H) A confocal time series image stack was acquired in the cytoplasm of a COOH-PS [YO] NP-treated HEp-2 cell (frame size: 512×512 pixels; pixel size: 0.03 mm; scan speed: 6.4 ms/pixel). One image of the resulting time series stack is shown. Note that the selected region also contains a region outside the cell (m, medium). (I) Two-dimensional spatial autocorrelation of the image stack (as described in B). Diffusion coefficient distribution maps of the region shown in (H) along with the respective diffusion coefficient *(D)* color maps were generated for the diffusion coefficient ranges between 0 and 2.7 µm2s-1 (J), or between 0 and 0.48 µm2s-1 (K). Black areas within the diffusion maps indicate regions not covered by the diffusion coefficient range displayed or areas with fits of insufficient quality. Bar, 10 µm.

To measure fast (diffusion) as well as slower (interactions) dynamic processes of NPs in cellular systems Raster Image Correlation Spectroscopy (RICS) was used complementary to FCS. RICS analyses intensity fluctuations between neighboring pixels by spatially autocorrelating the image in x and y direction using two-dimensional fast Fourier transformation [23]. RICS therefore has the additional advantage to provide spatial information of particle dynamics. Similar to FCS the measured autocorrelation function can be fitted to appropriate models to retrieve parameters for diffusion and concentration. In contrast to FCS, which is limited to a small volume, RICS additionally provides spatial information. As a control, RICS was first performed with fluorescent COOH-PS [YO] NPs in water at 25°C (Figure 5A). A representative correlation is shown as a one-dimensional map in Figure 5B. When overlapping regions of interest (ROIs) within the time series image stack are subjected to correlation, a diffusion map is created (Figure 5C). The measured diffusion coefficient of the COOH-PS [YO] NPs in water was on average 8 µm^2^s^−1^, which is in agreement with our FCS measurements (*D*_FCS_ = 8.1±1.1 µm^2^s^−1^).

RICS experiments were next performed in the nucleus of COOH-PS [YO] NP-loaded living HEp-2 cells, revealing a diffusion coefficient *D*_RICS_ between 0.3 and 1.7 µm^2^s^-1^ (Figure 5D-G). Considering the increased viscosity of the nucleoplasmic environment compared to water [34], these values translate into apparent hydrodynamic radii between ∼30 nm and ∼200 nm, again in excellent agreement with the FCS data (Figure 4E). Due to the lack of knowledge of the underlying nuclear architecture within the RICS imaging frame, we are currently unable to assign with certainty variations of the diffusion coefficient to potential diffusion barriers in the nucleus (i.e. nucleoli) or to the random appearance of differently sized small NP complexes. Since such variations were likewise observed *in vitro*, where no diffusion barriers exist, our RICS data suggest that COOH-PS [YO] NPs exist as a mixture of monodisperse and very small cluster populations moving homogenously throughout the nuclear volume by diffusion.

RICS analysis of nanoparticle diffusion was also performed in the cytoplasm (Figure 5H–K). In cytoplasmic regions with moderate COOH-PS [YO] NP-fluorescence, the diffusion coefficient ranged from below 0.05 µm^2^s^−1^ and 0.5 µm^2^s^−1^. In areas with high density of mitochondria, the diffusion coefficient was well below 0.1 µm^2^s^−1^, indicative of very slow diffusion and/or binding (Figure 5J and K). The diffusion coefficient of COOH-PS [YO] NPs in the medium, e.g. in the extracellular milieu, was significantly higher (*D*_RICS_>2 µm^2^s^−1^; Figure 5H and J; area marked with m). These data are fully consistent with our FRAP and FCS measurements and demonstrate the power of RICS to spatially resolve NP dynamics and bio-interactions inside and outside of living cells with high precision.

Due to an improved signal to noise ratio in confocal images compared to single point FCS measurements, *D* values obtained for NPs in living cells by RICS can be determined with higher accuracy [23], [42]. Notably, our data also reveal that RICS dynamics measurements of fluorescent particles within living cells can be done in the presence of large immobile features commonly found in the cell (i.e. mitochondria). The accessible timescales in RICS span all the way from microseconds to seconds [23], as evidenced by revealing a location-dependent 3 orders of magnitude difference in the diffusion coefficient of COOH-NPs in the cytoplasm (Figure 5J and K).

### Fast uptake of PS NPs into living cells

**Figure 6:**
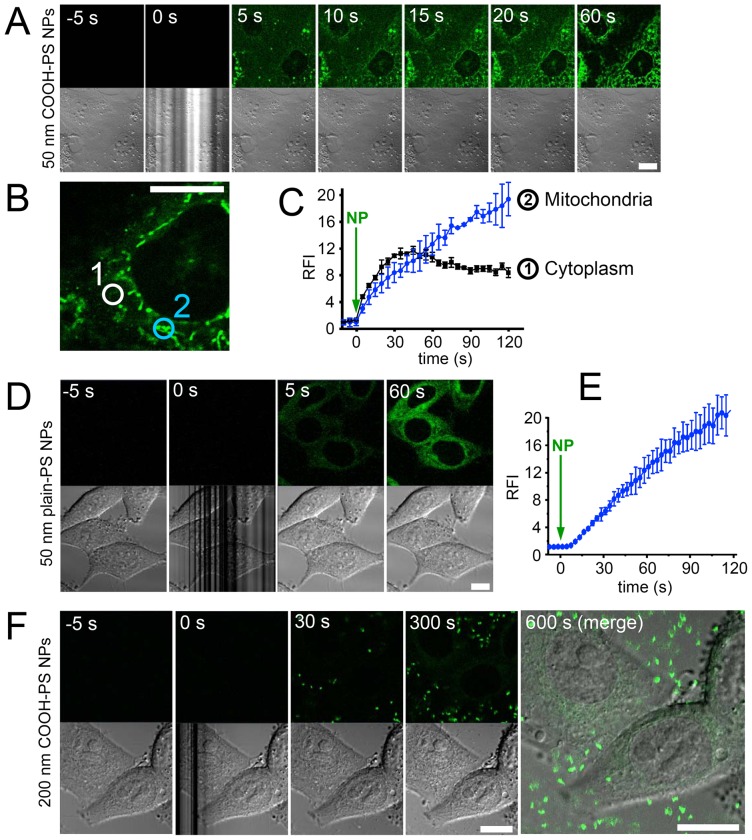
Rapid uptake of polystyrene nanoparticles in living cells. (A) Living HEp-2 cells were imaged to visualize particle fluorescence by confocal microscopy (upper panel) and differential interference contrast (DIC, lower panel). Fluorescent COOH-PS [YO] NPs were added directly to the culture medium at time point 0 seconds (0 s). Note, that the application of NPs is visualized by the distorted DIC image. (B) Circular regions of interest within the diffuse cytoplasmic pattern (1) or at mitochondria (2) were selected to measure the accumulation of fluorescent particles over time at different intracellular locations. (C) Quantification of measurements as described in (B) for COOH-PS [YO] NP fluorescence accumulation in the diffuse cytoplasm (black graph) and at mitochondria (blue graph). (D) Same experiment as in (A), using fluorescently labelled plain-PS [YG] NPs. (E) Quantification of measurements as described in (D) for plain-PS [YG] NP fluorescence accumulation in the cytoplasm. Relative fluorescence intensity (RFI) in C and E was normalized to 1 at 0 seconds. (F) Same experiment as in (A), using bulk sized (200 nm diameter) fluorescently labelled COOH-PS [YG] particles. Bars, 10 µm. NP, addition of nanoparticles; RFI, relative fluorescence intensity; s, seconds.

To investigate the dynamics of cellular NP uptake, confocal image series of living HEp-2 cells were recorded before, during and after addition of fluorescent PS NPs to the medium (Figure 6). Fluorescence of COOH-PS [YO] NPs was detectable in the cytoplasm as early as 5 seconds after their addition to the cells (Figure 6A). The subsequent time-lapse between 5 seconds and 1 minute is characterized by a steady increase of fluorescence intensity and accumulation of COOH-PS [YO] NPs in the cytoplasm and at distinct reticulated structures resembling mitochondria. The particle-fluorescence in the cytoplasm and at mitochondria was quantitated in representative subcellular regions of interest (Figure 6C). While accumulation of COOH-PS [YO] NPs in the cytoplasm reached a plateau after 30 seconds, accumulation at mitochondria continued to increase over several minutes (Figure 6C). Ten minutes after particle addition no further increase in cellular fluorescence was observed (data not shown). The distinct fluorescence pattern and rapid uptake was likewise observed in HeLa cells and mouse embryonic fibroblasts (data not shown), indicating that cytoplasmic PS NP wash-in in the seconds regimen is not cell-type specific. Applying the same imaging settings, particle fluorescence began to appear in the nucleus 3 to 4 minutes after particle application (data not shown). This 'delay' is consistent with our FRAP data that revealed a relatively slow movement/exchange of nanoparticles at the nuclear membrane (Figure 3D). We next tested PS particles with different surface characteristics, fluorochrome-labelling or size, and observed fast uptake of plain-PS [YG] NPs into living cells (Figure 6D and E), whereas bulk-sized COOH-PS [YG] particles with a diameter of 200 nm did not enter living HEp-2 cells during an observation time frame of 30 minutes (Figure 6F, and data not shown).

The time kinetics of PS NP uptake correspond to pathways such as macropinocytosis and clathrin-dependent or clathrin-independent endocytosis where intracellular vesicles are observed after a few seconds to 1.5 minutes [9], [43], [44]. Caveolae can be excluded as portals of PS NP entry because in most cells caveolae are only slowly internalized (halftime: 20 minutes) and the small vesicles (50–60 nm in diameter) carry little fluid-phase volume [9]. In fact, the observation of COOH-PS [YO] NP occurring in the cytoplasm within a few seconds after their addition to the cells reminds of rapid rates of endocytosis that have been demonstrated in nerve terminals with a time constant of τ = 1–2 seconds [24], [45]. While the underlying mechanisms are not yet fully understood, e.g. if the endocytotic vesicles require a clathrin coat or form in a coat-independent manner, such events definitely are dynamin-dependent [24].

### Dynasore inhibits rapid uptake of nanoparticles into living cells

**Figure 7:**
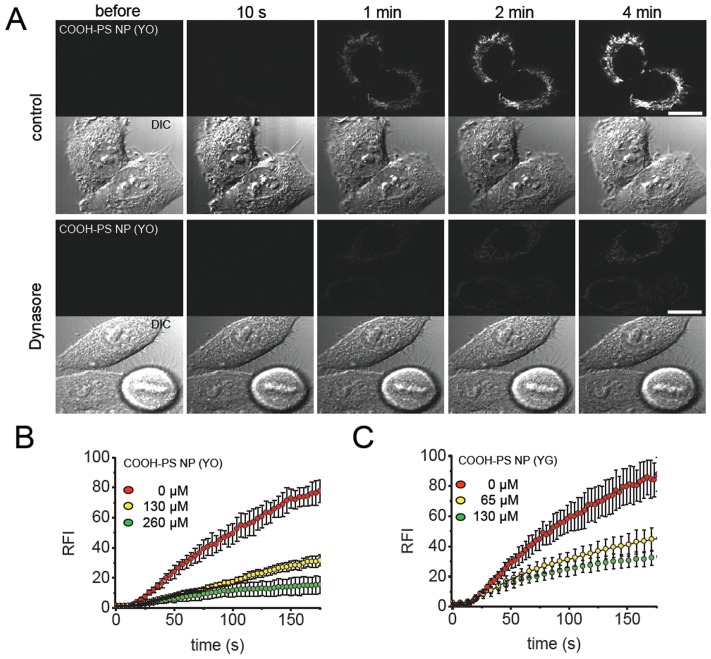
Dynasore inhibits uptake of COOH-PS NPs. (A) HEp-2 cells were incubated with fluorescent COOH-PS [YO] NPs in the absence (upper panel) or presence of 130 µM dynasore. Representative confocal images of fluorescence in mid-nucleus sections along with the DIC channel were acquired over time. (B) Experiments as in (A) were performed using different dynasore concentrations. Cellular fluorescence accumulation was quantified over time from at least 15 measurements each. (C) Same as in (B) using yellow-green [YG] labelled COOH-PS NPs. Bars, 10 µm. min, minutes; RFI, relative fluorescence intensity; s, seconds.

In order to further define the uptake mechanism a time-lapse of living HEp-2 cells was recorded before and after the application of fluorescent PS NPs to the medium in the presence of dynasore. Dynasore specifically inhibits the GTPase activity of dynamins, but does not interfere with other small GTPases [46]. Within 1–2 seconds after addition it blocks all dynamin-dependent endocytic pathways by capturing clathrin-coated or uncoated pits in various stages of vesicle formation before they pinch off the membrane [47]. Representative micrographs show that dynasore significantly inhibits the cytoplasmic uptake of COOH-PS [YO] NPs (Figure 7A). Quantification of intracellular NP-fluorescence shows a 4-fold or 8-fold reduction, respectively, of signal intensity that is correlated to an increase of inhibitor concentration (Figure 7B). Dynasore concentration-dependent decrease of cytoplasmic nanomaterial wash-in was likewise observed using differently labelled COOH-PS [YG] NPs (Figure 7C). Since macropinocytosis is dynamin-independent, the combination of our time kinetics and inhibitor results suggests that PS NPs enter cells by fast, clathrin-dependent or clathrin-independent endocytosis. The assumption of a clathrin-dependent portal for PS NP entry into cells is in agreement with a recent report showing that in a monocytic cell line anti-sense RNA-mediated depletion of dynamin II as well as clathrin results in significant inhibition of uptake of COOH- as well as amino-functionalized PS particles with a diameter of 100 nm [48]. However, it has to be noted that the analyses by Lunov et al. do not directly address fast uptake mechanisms, since they were performed somewhat delayed, e.g. >30 minutes after addition of the particles.

While PS as well as silica NPs rapidly traverse the cell membrane via dynamin-dependent endocytosis (Figure 7, and data not shown), COOH-PS [YO] NPs additionally showed rapid endosomal escape by accumulation at mitochondria within a minute. In contrast, plain-PS [YG] NPs recruit to and remain attached to vesicular structures in the perinuclear region. These results are consistent with the idea that modulation of NP-properties can be developed into controlled subcellular targeting.

## Conclusions

In this study we visualize the route of NP movement inside the living cell in a time and space resolved manner. A protocol of multimodal kinetic imaging is established that enables local and quantitative analyses of NP-behavior in live cells, e.g. a definition of the subcellular NP-bio-interface. Given the existing expertise in fluorochrome-labelling of NPs or their intrinsic fluorescent properties, such systematic kinetic imaging has the potential to develop a framework for definition of nanomaterial interactions at the cell membrane and within subcellular compartments of living cells. Our results show that location and interactions with cellular structures seem to correlate with distinct NP-characteristics, which implies that every (new) nanomaterial has to be tested individually. However, it can be assumed that groups of pathways and schemes of bio-interactions with common paradigms will emerge. Consistent with this concept, definition of individual cellular NP-networks may be instrumental in resolving as yet contradicting results concerning the fate of nanomaterials within living cells, and respective cellular end points.

Investigation of NP biophysics within living cells, as performed here, represents a prerequisite to better understand the molecular mechanisms of NP-bio-interactions, since this likewise requires quantitative analyses and observation of subcellular microenvironments at high resolution. Elucidation of NP delivery within cells is the basis of nanomaterial exposition in tissues and whole organisms, and therefore important for progress in both research areas, biomedical nanotechnology and nanotoxicology. Temporal and spatial positioning of NPs within the context of the living cellular environment is essential to develop subcellular targeting, e.g. clinical NP applications and nanomaterial design. Consistent with this we describe for the first time that PS and silica NPs generally act as monodisperse, highly dynamic entities within the cytoplasm and the cell nucleus, strengthening the concept of direct interactions between xenobiotic particles and cell components, e.g. cellular function.

## Materials and Methods

### Particles

Fluorescent particles: Carboxy-coated, yellow-orange polystyrene (COOH-PS [YO], 50 nm) NPs, carboxy-coated yellow-green polystyrene (COOH-PS [YG], 50 nm) NPs, plain yellow-green polystyrene (plain-PS [YG], 50 nm) NPs, and bulk carboxy-coated, yellow-green polystyrene (COOH-PS [YG], 200 nm) particles were from Polysciences. FITC-labelled silica NPs (50 nm) were purchased from Kisker. All particles were added directly to the cell culture medium at concentrations of 25 µg/ml. Each lot of NPs was subjected to *in vitro* analysis by FCS for the presence of free dye. FCS discriminates two (or more) fluorescent species of different size in the same solution by fitting the resulting autocorrelation functions with one-, two or multi-component diffusion models (see below). In all imaging experiments particle lots were used with FCS autocorrelation curves that could be fitted with one-component models of diffusion, e.g. diffusion times typical of the fluorescent NPs exclusive of free dye.

### Tem

Nanoparticle stock solutions were diluted with water and a small aliquot was placed on 400 mesh carbon-coated formvar copper grids. After 10 min, excess fluid was discarded and the samples were negatively stained with uranyl acetate (2%) in water for 30 seconds. Finally the specimens were examined with a transmission electron microscope CEM 902A (Zeiss Oberkochen) at an acceleration voltage amounting to 80 kV.

### Cell culture

HEp-2 and HeLa cells obtained from the American Tissue Culture Collection ATCC (Rockville, USA), and transformed mouse fibroblasts (NIH-3T3) from wild-type mice (a kind gift of Zhao-Qi Wang, FLI Jena) were cultured in Dulbecco's modified Eagle's medium (DMEM) supplemented with 10% fetal calf serum in a 10% CO_2_ atmosphere at 37°C. For live cell imaging experiments, cells were seeded on 42 mm glass dishes (Saur Laborbedarf, Reutlingen, Germany). Viability assays of NP (COOH-PS, plain-PS or silica)-loaded HEp-2, U-2 OS and NIH-3T3 cells did not reveal cell death or retarded cell growth behavior [17], [18] and data not shown. Consistent with unaltered cell viability, live cell imaging of cell division after intracellular COOH-PS NP-wash in appeared normal with no delays in mitotic events (Figure S1 A).

### Recombinant Proteins

Full-length green and red fluorescent protein were expressed as His_6_-tagged fusion proteins in *Escherichia coli* BL21(DE3) by PCR-cloning of full-length GFP and RFP cDNAs. Details on the construction of the expression plasmids pTOPO-EGFP-HisMyc and pTOPO-mRFP-HisMyc will be provided upon request. Expression and purification of recombinant proteins were performed according to protocols detailed previously [49]. Protein concentrations were determined using the micro BSA protein assay kit (Pierce).

### Frap

Fluorescence Recovery after Photobleaching (FRAP) experiments were carried out on a Zeiss LSM 510Meta or LSM 710 confocal microscope (Carl Zeiss, Jena, Germany) essentially as described before [50], [51]. Briefly, two to ten images were taken before the bleach pulse and 50–200 images after bleaching of circular 'regions of interest' (ROIs) at 0.05% laser transmission to minimize scan bleaching. Image acquisition frequency was adapted to the recovery rate of the respective fluorescence signal. The pinhole was adjusted to 1 airy unit. FRAP data were corrected for background signals and scan bleaching signal decay according to Rabut and Ellenberg [20]. Origin software (OriginLab, Northhampton, MA, USA) was used for non-linear regression fitting of FRAP curves. FRAP data were fitted with an exponential recovery function (Eq. 1) considering two exponential terms. 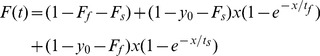
(1)with F(t): fluorescence at any time, F_f_: amount of fast fraction, F_s_: amount of slow fraction, y_0_: fluorescence intensity immediately after the bleach pulse, *t*_f_: rate constant of fast fraction, *t*_s_: rate constant of slow fraction.

Since FRAP for NPs measured in living cells always recovered to pre-bleach values it was not necessary to include an immobile fraction into the fitting function. The two exponential terms describe the behaviour of a fast and a slow fraction of molecules in the FRAP ROI as explained in detail elsewhere [52], [53]. Briefly, the fast fraction is assumed to be comprised of freely diffusing and randomly colliding molecules. This assumption is consistent with a very rapid fluorescence recovery in the first milliseconds after the bleach pulse. The second exponential term describes fluorescence recovery of molecules which exhibit potentially specific binding reactions at an immobile structure within the FRAP ROI [52], [53]. For evaluation of NP dynamics in Figure 3, only the fractions and recovery halftimes of the slower moving population was considered. This slower fraction is termed F*_(slow)_* in Figure 3. The recovery halftime (*t*_50%_) of the slow fraction was calculated as ln(2) × *t*_s_, as deduced previously by Bulinski et al. [52].

### Fcs

Fluorescence correlation spectroscopy (FCS) measurements were performed at 37°C on a LSM 510Meta/ConfoCor2 combi system using a C-Apochromat infinity-corrected 1.2 NA 40X water objective (Carl Zeiss, Jena, Germany) as described in detail before [54]. Briefly, NP fluorescence in vitro and in living cells was spot-illuminated with the 488 nm line of a 20 mW Argon laser at 5.5 Ampere tube current attenuated by an acousto-optical tunable filter (AOTF) to 0.1%. The detection pinhole had a diameter of 70 µm and emission was recorded through a 505–530 nm band-path filter. For the measurements, 30 time series of 10 seconds (s) each were recorded with a time resolution of 1 µs and then superimposed for fitting to a one-component-free or one-component-anomalous diffusion models in three dimensions with triplet function using Origin Software (OriginLab, Northhampton, MA, USA) or ZEN software (Carl Zeiss, Jena, Germany). Measurements were repeated at least 10 times to determine mean values and standard deviations. The calibration of the focal volume dimensions was done with Rhodamine-6G (R6G) (Sigma) dissolved in water, which has a diffusion coefficient of *D*_R6G_ = 420 µm^2^ s^−1^ [55].

### Determination of the hydrodynamic radius

According to Stokes law, the diffusion coefficient *D* for spherical molecules is inversely proportional to the hydrodynamic radius *R_h_*, determined by:
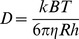
(2)with the temperature T, Boltzmann constant *k_B_* and the viscosity *η*.

### Rics

Raster scanning image correlation spectroscopy (RICS) was performed as described previously [55] on a LSM 710 (Carl Zeiss MicroImaging GmbH, Jena) using a C-Apochromat infinity-corrected 1.2 NA 40X water objective, a frame size of 512×512 pixels, a pixel size of 0.03 µm, and a scan speed of 6.4 µs/pixel. For RICS, cells were maintained in HEPES-buffered medium without phenol red to minimize background fluorescence. A time series of 50 images was recorded. After subtracting a moving average to remove slow moving structures and cellular movement the average spatial correlation was computed and fitted to a free 3-dimensional diffusion model provided by the inbuilt software module. The diffusion model and the spatial autocorrelation fit function are described in detail in Digman et al. [23], [42]. Diffusion maps were created using a region of interest (ROI) size of 64×64 pixels with 32 pixel shifts in each direction and the same model for fitting.

## Supporting Information

Figure S1 **Figure S1 shows subcellular localization of polystyrene nanoparticles throughout mitosis and their colocalization with mitochondrial structures in interphase cells.** (A, B) Living HEp-2 cells were incubated simultaneously with fluorescent COOH-PS [YO] NPs (red) and plain-PS [YG] NPs. Mid-nucleus confocal sections were acquired in (A) an interphase cell or (B) a mitotic cell. (C) Living HEp-2 cells were co-labelled with Mitotracker (green) and fluorescent COOH-PS [YO] NPs (red). A representative mid-nucleus confocal section was acquired in an interphase cell. (D) COOH-PS [YO] nanoparticles do not interfere with cell division. Living HEp-2 cells were incubated with fluorescent COOH-PS [YO] NPs. A representative cell in metaphase was selected for time lapse microscopy and followed through mitosis until the early G1 phase. Images show mid-nucleus confocal sections of particle fluorescence (green, inverted color) and differential interference contrast (DIC). Bars, 10 µm. min, minutes.
(TIF)

Table S1 **Table S1 provides particle characterization by dynamic light scattering analysis.** Particle size and zeta potential were measured using the Zetasizer Nano-ZS (Malvern Instruments Ltd). The mean size of polystyrene or silica NPs were measured by dynamic light scattering. The zeta potential was measured by laser Doppler electrophoresis. FITC, fluorescein isothiocyanate; mV, millivolt; nm, nanometer; PS, polystyrene; YG, yellow green; YO, yellow orange; ζ, zeta.
(DOC)

## References

[1] Savic R, Luo L, Eisenberg A, Maysinger D (2003) Micellar nanocontainers distribute to defined cytoplasmic organelles. Science 300: 615–618. 12714738 10.1126/science.1078192

[2] Elder A, Oberdörster G (2006) Translocation and effects of ultrafine particles outside of the lung. Clin Occup Environ Med 5: 785–796. 17110292 10.1016/j.coem.2006.07.003

[3] Mortensen LJ, Oberdörster G, Pentland AP, Delouise LA (2008) In vivo skin penetration of quantum dot nanoparticles in the murine model: the effect of UVR. Nano Lett 8: 2779–2787. 18687009 10.1021/nl801323y PMC4111258

[4] Canton I, Battaglia G (2012) Endocytosis at the nanoscale. Chem Soc Rev 41: 2718–2739. 22389111 10.1039/c2cs15309b

[5] Park S, Hamad-Schifferli K (2010) Nanoscale interfaces to biology. Curr Opin Chem Biol 14: 616–622. 20674473 10.1016/j.cbpa.2010.06.186 PMC2953582

[6] Stark WJ (2011) Nanoparticles in biological systems. Angew Chem Int Ed Engl 50: 1242–1258. 21290491 10.1002/anie.200906684

[7] Moyano DF, Rotello VM (2011) Nano meets biology: structure and function at the nanoparticle interface. Langmuir 27: 10376–10385. 21476507 10.1021/la2004535 PMC3154611

[8] Nel A, Mädler L, Velegol D, Xia T, Hoek EM, et al (2009) Understanding biophysicochemical interactions at the nano-bio interface. Nat Mater 8: 543–557. 19525947 10.1038/nmat2442

[9] Conner SD, Schmid SL (2009) Regulated portals of entry into the cell. Nature 422: 37–44. 10.1038/nature01451 12621426

[10] Doherty GJ, McMahon HT (2009) Mechanisms of endocytosis. Annu Rev Biochem 78: 857–902. 19317650 10.1146/annurev.biochem.78.081307.110540

[11] Goodsell DS (2011) Miniseries: Illustrating the machinery of life: Eukaryotic cell panorama. Biochem Mol Biol Educ 39: 91–101. 21445900 10.1002/bmb.20494

[12] Dix JA, Verkman AS (2008) Crowding effects on diffusion in solutions and cells. Annu Rev Biophys 37: 247–263. 18573081 10.1146/annurev.biophys.37.032807.125824

[13] Cedervall T, Lynch I, Lindman S, Berggård T, Thulin E, et al (2007) Understanding the nanoparticle-protein corona using methods to quantify exchange rates and affinities of proteins for nanoparticles. Proc Natl Acad Sci USA 104: 2050–2055. 17267609 10.1073/pnas.0608582104 PMC1892985

[14] Suh J, Wirtz D, Hanes J (2003) Efficient active transport of gene nanocarriers to the cell nucleus. Proc Natl Acad Sci USA 100: 3878–3882. 12644705 10.1073/pnas.0636277100 PMC153016

[15] Cambi A, Lidke DS, Arndt-Jovin DJ, Figdor CG, Jovin T (2007) Ligand-Conjugated Quantum Dots Monitor Antigen Uptake and Processing by Dendritic Cells. Nano Lett 7: 970–977. 17388641 10.1021/nl0700503

[16] Xia T, Kovochich M, Brant J, Hotze M, Sempf J, et al (2006) Comparison of the abilities of ambient and manufactured nanoparticles to induce cellular toxicity according to an oxidative stress paradigm. Nano Lett 6: 1794–1807. 16895376 10.1021/nl061025k

[17] Xia T, Kovochich M, Liong M, Zink JI, Nel AE (2008) Cationic polystyrene nanosphere toxicity depends on cell-specific endocytic and mitochondrial injury pathways. ACS Nano 2: 85–96. 19206551 10.1021/nn700256c

[18] Chen M, von Mikecz A (2005) Formation of nucleoplasmic protein aggregates impairs nuclear function in response to SiO_2_ nanoparticles. Exp Cell Res 305: 51–62. 15777787 10.1016/j.yexcr.2004.12.021

[19] Chen M, Singer L, Scharf A, von Mikecz A (2008) Nuclear polyglutamine-containing protein aggregates as active proteolytic centers. J Cell Biol 180: 697–704. 18283109 10.1083/jcb.200708131 PMC2265588

[20] Rabut G, Ellenberg J (2005) Photobleaching techniques to study mobility and molecular dynamics of proteins in live cells: FRAP, iFRAP, and FLIP. In Goldman RD, Spector DL, editors. Live Cell Imaging: A Laboratory Manual. Cold Spring Harbor, New York, Cold Spring Harbor Laboratory Press.101–126. 10.1101/pdb.top90 21123431

[21] Magde D, Elson E, Webb WW (1972) Thermodynamic fluctuations in a reacting system - measurement by Fluorescence Correlation Spectroscopy. Phys Rev Lett 29: 705–708.

[22] Bacia K, Kim SA, Schwille P (2006) Fluorescence cross-correlation spectroscopy in living cells. Nat Methods 3: 83–89. 16432516 10.1038/nmeth822

[23] Digman MA, Brown CM, Sengupta P, Wiseman PW, Horwitz AR, et al (2005) Measuring fast dynamics in solutions and cells with a laser scanning microscope. Biophys J 89: 1317–1327. 15908582 10.1529/biophysj.105.062836 PMC1366616

[24] Smith SM, Renden R, von Gersdorff H (2008) Synaptic vesicle endocytosis: fast and slow modes of membrane retrieval. Trends Neurosc 31: 559–568. 10.1016/j.tins.2008.08.005 PMC3626563 18817990

[25] Pluskota A, Horzowski E, Bossinger O, von Mikecz A (2009) In Caenorhabditis elegans nanoparticle-bio-interactions become transparent: silica-nanoparticles induce reproductive senescence. PLoS One 4: e6622. 19672302 10.1371/journal.pone.0006622 PMC2719910

[26] Song Y, Li X, Wang L, Meighan T, Castranova V, et al (2011) Nanomaterials in humans: identification, characteristics, and potential damage. Toxicol Pathol 39: 841–849. 21768271 10.1177/0192623311413787 PMC4706159

[27] Chithrani BD, Chan WC (2007) Elucidating the mechanism of cellular uptake and removal of protein-coated gold nanoparticles of different sizes and shapes. Nano Lett 7: 1542–1550. 17465586 10.1021/nl070363y

[28] Cheng J, Fernando KA, Veca LM, Sun YP, Lamond AI, et al (2008) Reversible accumulation of PEGylated single-walled carbon nanotubes in the mammalian nucleus. ACS Nano 2: 2085–2094. 19206455 10.1021/nn800461u

[29] Huang JG, Leshua T, Gu FX (2011) Emerging nanomaterials for targeting subcellular organelles. Nano Today 6: 478–492.

[30] Nabeshi H, Yoshikawa T, Matsuyama K, Nakazato Y, Matsuo K, et al (2011) Systemic distribution, nuclear entry and cytotoxicity of amorphous nanosilica following topical application. Biomaterials 32: 2713–2724. 21262533 10.1016/j.biomaterials.2010.12.042

[31] Köster M, Frahm T, Hauser H (2005) Nucleocytoplasmic shuttling revealed by FRAP and FLIP technologies. Curr Opin Biotechnol 16: 28–34. 15722012 10.1016/j.copbio.2004.11.002

[32] Panté N, Kann M (2002) Nuclear pore complex is able to transport macromolecules with diameters of about 39 nm. Mol Biol Cell 13: 425–434. 11854401 10.1091/mbc.01-06-0308 PMC65638

[33] Hancock R (2004) A role for macromolecular crowding effects in the assembly and function of compartments in the nucleus. J Struct Biol 146: 281–290. 15099570 10.1016/j.jsb.2003.12.008

[34] Seksek O, Biwersi J, Verkman AS (1997) Translational diffusion of macromolecule-sized solutes in cytoplasm and nucleus. J Cell Biol 138: 131–142. 9214387 10.1083/jcb.138.1.131 PMC2139942

[35] Mastro AM, Babich MA, Taylor WD, Keith AD (1984) Diffusion of a small molecule in the cytoplasm of mammalian cells. Proc Natl Acad Sci USA 81: 3414–3418. 6328515 10.1073/pnas.81.11.3414 PMC345518

[36] Luby-Phelps K, Castle PE, Taylor DL, Lanni F (1987) Hindered diffusion of inert tracer particles in the cytoplasm of mouse 3T3 cells. Proc Natl Acad Sci USA 84: 4910–4913. 3474634 10.1073/pnas.84.14.4910 PMC305216

[37] Weiss M, Elsner M, Kartberg F, Nilsson T (2004) Anomalous subdiffusion is a measure for cytoplasmic crowding in living cells. Biophys J 87: 3518–3524. 15339818 10.1529/biophysj.104.044263 PMC1304817

[38] Guigas G, Weiss M (2008) Sampling the cell with anomalous diffusion - the discovery of slowness. Biophys J 94: 90–94. 17827216 10.1529/biophysj.107.117044 PMC2134854

[39] Lepock JR, Cheng KH, Campbell SD, Kruuv J (1983) Rotational diffusion of TEMPONE in the cytoplasm of Chinese hamster lung cells. Biophys J 44: 405–412. 6318842 10.1016/S0006-3495(83)84314-8 PMC1434848

[40] Bacia K, Schwille P (2003) A dynamic view of cellular processes by in vivo fluorescence auto- and cross-correlation spectroscopy. Methods 29: 74–85. 12543073 10.1016/s1046-2023(02)00291-8

[41] Gennerich A, Schild D (2002) Anisotropic diffusion in mitral cell dendrites revealed by fluorescence correlation spectroscopy. Biophys J 83: 510–522. 12080138 10.1016/S0006-3495(02)75187-4 PMC1302165

[42] Digman MA, Gratton E (2009) Analysis of diffusion and binding in cells using the RICS approach. Microsc Res Tech 72: 323–332. 19067357 10.1002/jemt.20655 PMC4364519

[43] Artalejo CR, Elhamdani A, Palfrey HC (2002) Sustained stimulation shifts the mechanism of endocytosis from dynamin-1-dependent rapid endocytosis to clathrin- and dynamin-2-mediated slow endocytosis in chromaffin cells. Proc Natl Acad Sci USA 99: 6358–6363. 11959911 10.1073/pnas.082658499 PMC122953

[44] Hacker U, Albrecht R, Maniak M (1997) Fluid-phase uptake by macropinocytosis in Dictyostelium. J Cell Sci 110: 105–112. 9044041 10.1242/jcs.110.2.105

[45] von Gersdorff H, Matthews G (1994) Inhibition of endocytosis by elevated internal calcium in a synaptic terminal. Nature 370: 652–655. 8065451 10.1038/370652a0

[46] Macia E, Ehrlich M, Massol R, Boucrot E, Brunner C, et al (2006) Dynasore, a cell-permeable inhibitor of dynamin. Dev Cell 10: 839–850. 16740485 10.1016/j.devcel.2006.04.002

[47] Kirchhausen T, Macia E, Pelish HE (2008) Use of dynasore, the small molecule inihibtor of dynamin, in the regulation of endocytosis. Methods Enzymol 438: 77–93. 18413242 10.1016/S0076-6879(07)38006-3 PMC2796620

[48] Lunov O, Syrovets T, Loos C, Beil J, Delacher M, et al (2011) Differential uptake of functionalized polystyrene nanoparticles by human macrophages and a monocytic cell line. ACS Nano 5: 1657–1669. 21344890 10.1021/nn2000756

[49] Hemmerich P, Stoyan T, Wieland G, Koch M, Lechner J, et al (2000) Interaction of yeast kinetochore proteins with centromere-protein/transcription factor Cbf1. Proc Natl Acad Sci USA 97: 12583–12588. 11070082 10.1073/pnas.97.23.12583 PMC18807

[50] Schmiedeberg L, Weisshart K, Diekmann S (2004) Meyer zu Hoerste, G.; Hemmerich, P. High- and low-mobility populations of HP1 in heterochromatin of mammalian cells. Mol Biol Cell 15: 2819–2833. 15064352 10.1091/mbc.E03-11-0827 PMC420105

[51] Weidtkamp-Peters S, Lenser T, Negorev D, Gerstner N, Hofmann TG, Schwanitz G, et al (2008) Dynamics of component exchange at PML nuclear bodies. J Cell Sci 121: 2731–2743. 18664490 10.1242/jcs.031922

[52] Bulinski JC, Odde DJ, Howell BJ, Salmon TD, Waterman-Storer CM (2001) Rapid dynamics of the microtubule binding of ensconsin in vivo. J Cell Sci 114: 3885–3897. 11719555 10.1242/jcs.114.21.3885

[53] Sprague BL, Pego RL, Stavreva DA, McNally JG (2004) Analysis of binding reactions by fluorescence recovery after photobleaching. Biophys J 86: 3473–4395. 15189848 10.1529/biophysj.103.026765 PMC1304253

[54] Weidtkamp-Peters S, Weisshart K, Schmiedeberg L, Hemmerich P (2009) Fluorescence correlation spectroscopy to assess the mobility of nuclear proteins. Methods Mol Biol 464: 321–341. 18951193 10.1007/978-1-60327-461-6_18

[55] Petrásek Z, Schwille P (2008) Precise measurement of diffusion coefficients using scanning fluorescence correlation spectroscopy. Biophys J 94: 1437–1448. 17933881 10.1529/biophysj.107.108811 PMC2212689

