# Supplementary figures and images for: Defining the Subcellular Interface of Nanoparticles by Live-Cell Imaging
Source: PLoS One. 2013 Apr 26;8(4):e62018. doi: 10.1371/journal.pone.0062018 (PMC3637372; doi:10.1371/journal.pone.0062018)

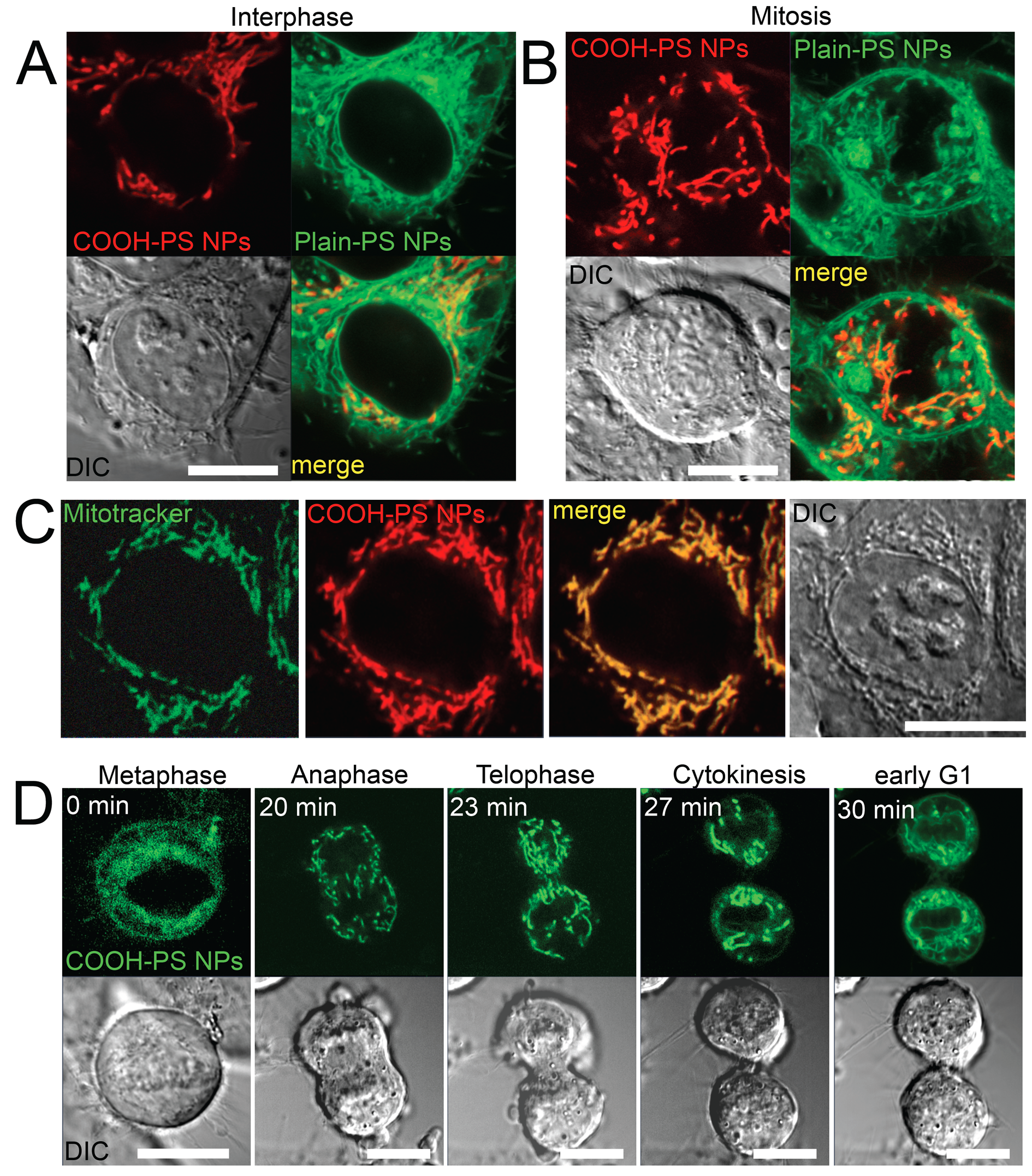

Supplement: Figure S1 — shows subcellular localization of polystyrene nanoparticles throughout mitosis and their colocalization with mitochondrial structures in interphase cells. (A, B) Living HEp-2 cells were incubated simultaneously with fluorescent COOH-PS [YO] NPs (red) and plain-PS [YG] NPs. Mid-nucleus confocal sections were acquired in (A) an interphase cell or (B) a mitotic cell. (C) Living HEp-2 cells were co-labelled with Mitotracker (green) and fluorescent COOH-PS [YO] NPs (red). A representative mid-nucleus confocal section was acquired in an interphase cell. (D) COOH-PS [YO] nanoparticles do not interfere with cell division. Living HEp-2 cells were incubated with fluorescent COOH-PS [YO] NPs. A representative cell in metaphase was selected for time lapse microscopy and followed through mitosis until the early G1 phase. Images show mid-nucleus confocal sections of particle fluorescence (green, inverted color) and differential interference contrast (DIC). Bars, 10 µm. min, minutes. (TIF) [file pone.0062018.s001.tif]
