# Supplementary material for: Defining the Subcellular Interface of Nanoparticles by Live-Cell Imaging
Source: PLoS One. 2013 Apr 26;8(4):e62018. doi: 10.1371/journal.pone.0062018 (PMC3637372; doi:10.1371/journal.pone.0062018)
Supplement: Table S1 — provides particle characterization by dynamic light scattering analysis. Particle size and zeta potential were measured using the Zetasizer Nano-ZS (Malvern Instruments Ltd). The mean size of polystyrene or silica NPs were measured by dynamic light scattering. The zeta potential was measured by laser Doppler electrophoresis. FITC, fluorescein isothiocyanate; mV, millivolt; nm, nanometer; PS, polystyrene; YG, yellow green; YO, yellow orange; ζ, zeta. (DOC) [file pone.0062018.s002.doc]

**Supplemental Table 1.** Particle characterization by dynamic light scattering analysis.

Particle Diameter [nm] Zeta potential, ζ [mV]

______________________________________________________________________

COOH-PS (YO), 50 nm 42.22 -54.6

COOH-PS (YG), 50 nm 50.54 -63.8

COOH-PS (YG), 200 nm 209.2 -69.8

plain-PS (YG), 50 nm 50.55 -78.8

silica (FITC), 50 nm 45.7 -48.7

______________________________________________________________________

Particle size and zeta potential were measured using the Zetasizer Nano-ZS (Malvern Instruments Ltd). The mean size of polystyrene or silica NPs were measured by dynamic light scattering. The zeta potential was measured by laser Doppler electrophoresis. FITC, fluorescein isothiocyanate; mV, millivolt; nm, nanometer; PS, polystyrene; YG, yellow green; YO, yellow orange; ζ, zeta.
